# An Unusual Presentation of Primary B-cell Lymphoma of the Small Bowel in an Immunosuppressant-Naive Patient With Newly Diagnosed Crohn’s Disease

**DOI:** 10.7759/cureus.40289

**Published:** 2023-06-12

**Authors:** William H Arata, Shauna Maty, Alexander Ferrera, Joseph Lieber

**Affiliations:** 1 Internal Medicine, Elmhurst Hospital Center, New York City, USA; 2 Anesthesiology, Elmhurst Hospital Center, New York City, USA; 3 Anesthesiology, Mt. Sinai Morningside/West, Manhattan, USA; 4 Internal Medicine/Nephrology, Elmhurst Hospital Center, New York City, USA

**Keywords:** immunosuppressant therapy, malignant lymphoma, inflammatory bowel disease, epidural b-cell lymphoma, crohn’s disease

## Abstract

Inflammatory bowel disease (IBD) consists of two primary conditions: ulcerative colitis (UC) and Crohn's disease (CD). UC primarily impacts the colon, leading to inflammation of the mucosal layer. Conversely, CD involves transmural inflammation and can affect any segment of the gastrointestinal tract, ranging from the oral cavity to the perianal region. Patients with CD can have symptoms for many years prior to diagnosis, or they may present acutely. We present the case of a 31-year-old male with a recent CD diagnosis and otherwise, no past medical history presenting with a week-long history of bilateral lower extremity swelling that started in the thighs and progressed downward, accompanied by a heavy sensation in the legs and intermittent numbness. Less than 24 hours into his hospital course, the patient experienced progressive bilateral numbness, saddle anesthesia, and urinary incontinence. Subsequently, the patient was taken for STAT MRI and emergent neurosurgery to alleviate the spinal cord compression and remove/biopsy a mass at the T6-T7 level that was later defined as a B-cell lymphoma. Our objectives are to describe the etiology of IBD complicated by lymphoma, to analyze the association between IBD and lymphoma, and to investigate the role that immunosuppressants play in the development of lymphoma from IBD, which we achieve through retrospective case analysis and associated literature review on symptom constellation. There is good evidence that malignant lymphoma of the bowel is a rare but significant complication of IBD in immunosuppressant-naive patients, apparently being more common in chronic UC. We suggest increased surveillance for this disease in immunosuppressant-naive patients, as the prognosis of lymphoma depends on the time of diagnosis.

## Introduction

There is a known association between patients with inflammatory bowel disease (IBD) receiving immunosuppressant therapy (azathioprine +/- 6-mercaptopurine) and developing non-Hodgkin lymphoma [1]. However, the association between specifically Crohn's disease (CD) and non‐Hodgkin's lymphoma remains controversial as previous studies of lymphoma risk in IBD have been underpowered to establish a definitive relationship and are typically not representative of lymphoma risk in IBD today [1]. Intestinal lymphoma generally arises in long‐standing CD in locations where the inflammatory disease is highly active [1]. This paper presents a case of a 31-year-old male patient with a CD with no previous history of immunosuppressants who presented with a metastatic B-cell intestinal lymphoma.

## Case presentation

We present the case of a 31-year-old male with newly diagnosed (not yet treated) CD who presented to the emergency department with upper back spasms and bilateral lower extremity swelling starting in the thighs and intermittent numbness. The patient reported 8/10 spasming back pain in the upper right side of his back; he postulated it was something he had done at the gym one week ago because the pain felt similar to another episode. The patient visited his chiropractor for back pain but said the manipulation exacerbated the spasms. The bilateral lower extremity swelling began six days prior. The patient reports he first noticed it in his thighs and then it started progressing downward, accompanied by intermittent numbness and no urinary incontinence.

**Figure 1:**
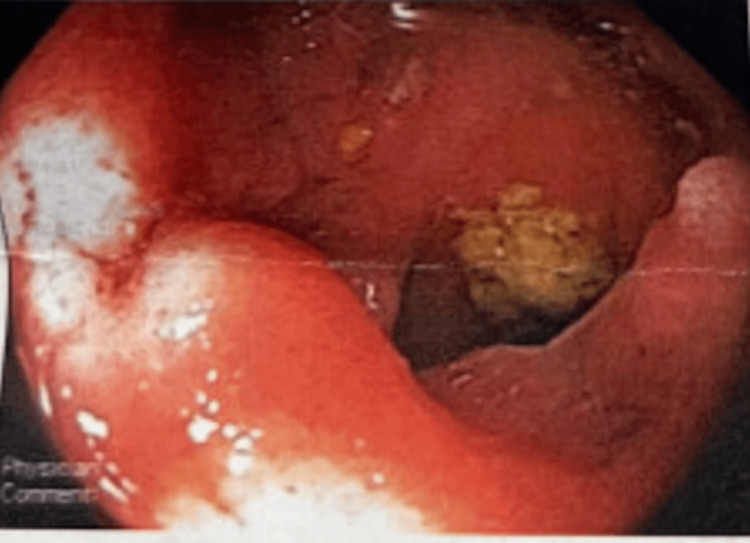
Submucosal lesions in the terminal ileum visible on colonoscopy

**Figure 2:**
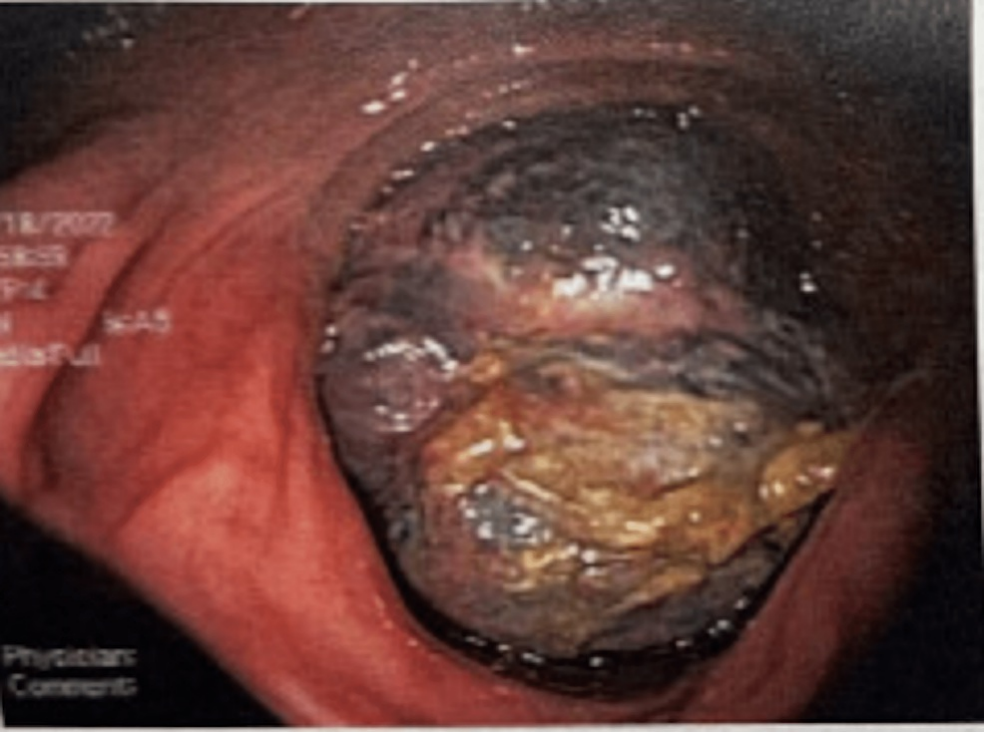
Malignant-appearing mass in the hepatic flexure

Notably, the patient is a healthy adult male who exercises frequently and has no significant past medical history and was otherwise in a normal state of health until one month ago when he presented to the hospital with progressive, severe upper abdominal pain for three weeks. At that time, he underwent a colonoscopy which showed multiple submucosal ulcerations in the terminal ileum (Figure 1) and a malignant-appearing mass in the hepatic flexure (Figure 2). The patient was given a follow-up appointment with the plan to start immunosuppressants at the next appointment eventually.

**Figure 3:**
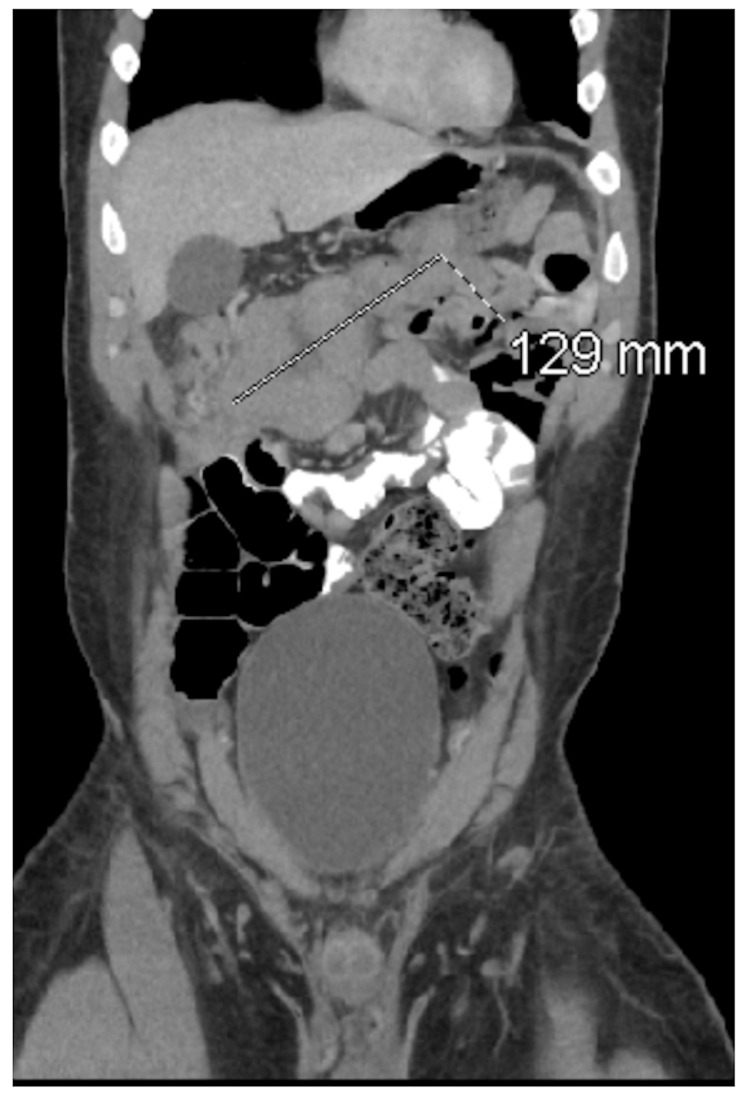
CT with contrast with coronal view showing a 12.9 cm mass at the hepatic flexure

Vitals upon this hospital admission: blood pressure 125/78 mmHg, heart rate 100 bpm, temperature 98.4F, SpO_2_ 99% on room air. HIV screening was negative. The patient received a CT abdomen pelvis with contrast, revealing a 12.9 cm mass at the hepatic flexure (Figure 3).

**Figure 4:**
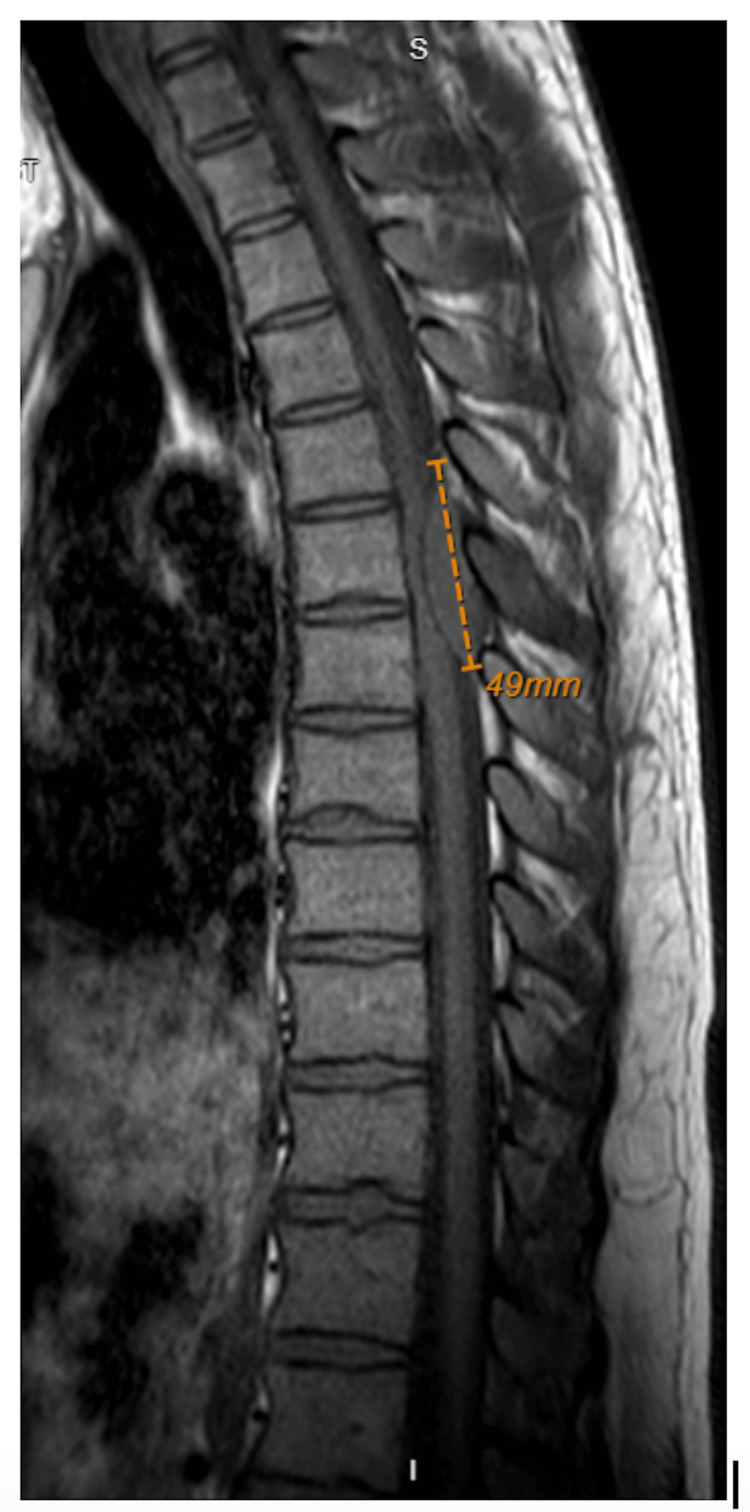
Thoracic MRI with sagittal view showing a 4.9 cm posterior epidural mass at T6-T7

It was unclear if the mass was secondary to Crohn’s flare or another etiology, and further imaging was recommended. The lower extremity swelling was hypothesized to be secondary to this mass compressing the inferior vena cava. The patient had sensation in the legs and was able to move them, though he reports they were feeling “heavy” from the fluid buildup and had intermittent episodes of numbness in the bilateral lower legs. Throughout this time, the patient’s main concern was the back pain and spasming; he was managed with dexamethasone 10mg, lidocaine 5% patch, and baclofen 5mg. Hours later in the evening, the patient’s numbness and weakness became more widespread in the lower extremities with diminished strength testing of his bilateral lower extremities (0/5). This was accompanied by saddle anesthesia and episodes of urinary incontinence. Neurology and neurosurgery were emergently consulted at this time. Emergent thoracic MRI showed a 4.9cm mass at the posterior spinal canal at T6-T7 (Figure 4).

Given his constellation of progressing symptoms and significant spinal cord involvement, the patient was recommended for emergent neurosurgery. The patient underwent a laminectomy of T6-T7 and was later found to be diagnosed with Burkitt's lymphoma.

## Discussion

The association between IBD and lymphoma is uncertain. A recent meta-analysis showed a marked increase in relative risk for developing lymphoma after a diagnosis of IBD [2]. In this study, they found an increased risk of developing myeloid leukemia in patients that were diagnosed with ulcerative colitis (UC). The risk of developing lymphoma in CD was unclear but, a modest risk of developing unspecified lymphoma couldn't be ruled out. However, this study could have been more extensive in that they could not ask for pharmacotherapy data. The disease duration appears as a possible independent risk factor for lymphoma [3]. A likely explanation of the pathogenesis of IBD-associated intestinal lymphoma is local sites of inflammation that may be particularly at risk of lymphoproliferation due to chronic B-cell stimulation [3]. Another possible factor in the genesis of lymphoma in IBD patients is immunosuppressants. There is a known association between receiving immunosuppressants for IBD and developing primary malignant lymphoma; however, the risk is shallow [4]. A meta‐analysis by Farrell et al. showed a fourfold increased risk of non‐Hodgkin's lymphoma in a subgroup of IBD patients treated with azathioprine and/or 6‐mercaptopurine [5]. An immunological mechanism could also explain this association with IBD and B-cell lymphoma. A study by Auer et al. showed that patients with an inactive CD of short duration and without previous treatment have relative and absolute B lymphocytosis [6]. The absolute T cell counts were likewise increased in this group. This increase in immune system reactivity and the resulting inflammation from the lymphocytosis could explain the association between CD and lymphoma risk.

Most patients with a diagnosis of lymphoma associated with IBD described in the literature were on long-term immunosuppressant therapy, and this association has been well-researched [7]. However, our patient had no history of immunosuppressant usage for his Crohn’s. Within a month of his Crohn’s diagnosis, he was diagnosed with a malignant B-cell intestinal lymphoma. Shepherd et al. reported seven cases of malignant lymphoma of the colon and rectum associated with UC and three cases related to CD in patients, not on immunosuppressant therapy [8]. Immunosuppressant therapy is the standard of care for patients with moderate-severe stage CD. These patients likely did not start therapy at all for their IBD prior to their diagnosis of malignant B-cell lymphoma due to various reasons not explained by the study.

In the same study, Sheperd et al. reported that the lymphomas also seemed to arise at the location of the active CD inflammation in the viscera [8]. Our patient had immunosuppressant-naive lymphoma likely at the site of inflammation of his CD found in the same month as his diagnosis of Crohn’s, highlighting an unusual case as the majority of Crohn’s-associated malignant lymphomas are associated with immunosuppressant usage. A similar case was found on review that also had an unusual presentation. This case described a 49-year-old man who presented with a several-month history of melena, and unintentional weight loss and was found on endoscopy to have erythematous mucosa at the jejunum suggestive of CD [9]. A biopsy was taken at the site and the patient was found to be positive for diffuse large B-cell lymphoma (DLBCL) [9]. This patient was also found to be immunosuppressant-naive further highlighting a likely association between the site of inflammation and the site of lymphoma development.

## Conclusions

There is good evidence that malignant lymphoma of the bowel is a rare but significant complication of IBD in immunosuppressant-naive patients, apparently being more common in chronic UC. We suggest increased surveillance for this disease in immunosuppressant-naive patients presenting with the symptomatology of IBD, as the prognosis of lymphoma depends on the time of diagnosis.
